# Delayed Recognition of Severe Systemic Envenomation after Copperhead Bite: A Case Report

**DOI:** 10.5811/cpcem2022.6.56592

**Published:** 2022-08-08

**Authors:** Patrick E. Kelly, Charles J. Gerardo

**Affiliations:** Duke University Hospital, Department of Surgery, Durham, North Carolina

**Keywords:** case report, snakebite, copperhead, Agkistrodon contortrix, antivenin

## Abstract

**Introduction:**

We report a case of severe systemic copperhead, *Agkistrodon contortrix*, envenomation that resulted in long-term sequelae.

**Case Report:**

A 72-year-old man presented to the emergency department after suffering a copperhead snakebite. He developed severe systemic toxicity before local tissue injury developed. Clinicians did not initially recognize his envenomation syndrome and sought alternative explanations for his systemic symptoms before polyvalent immune fab (ovine) antivenom was administered. Although the patient improved, he was discharged with new stage three chronic kidney disease.

**Conclusion:**

Although rare, copperhead envenomation can cause severe systemic toxicity. Envenomation should be promptly treated with antivenom.

## INTRODUCTION

The Crotalinae subfamily of snakes is responsible for 98% of the nearly 9,000 venomous bites in the United States per year.^1^,^2^ Envenomation can present with systemic toxicity, hemotoxicity, and venom-induced tissue injury. Patients bitten by the species *Agkistrodon contortrix* (copperheads) rarely develop severe systemic envenomation, and some authors have discouraged antivenom administration.^3^,^4^ We report a case in which this did occur and diagnosis as well as treatment were delayed to due to an atypical presentation for this species.

## CASE REPORT

A 72-year-old man with a history of hypertension treated with amlodipine and lisinopril presented to the emergency department (ED) within 30 minutes of copperhead snakebite to the right lateral ankle. A physician with expertise in snakebite and local snake identification verified the species by a photo of the offending snake. Initial vital signs were a temperature 36.7ºC, heart rate 100 beats per minute (bpm), respiratory rate 19 breaths per minute, blood pressure 144/74 millimeters of mercury (mm Hg), and oxygen saturation 95% on room air. Examination revealed puncture wounds (Image) without surrounding erythema, edema, or tenderness. The triage physician felt that the patient had suffered a dry bite and placed him in a room for further evaluation.

The patient then told his treating physician that he had also experienced chest tightness, shortness of breath, abdominal pain, and anxiety after the bite. Two hours after arrival to the ED, he developed nausea with repeated vomiting and diarrhea. He became lightheaded and lost consciousness in front of his wife. His physicians were called to the room. He was confused, and his vital signs were unstable with sinus bradycardia (heart rate 44 bpm), hypotension (blood pressure 74/52 millimeters of mercury), and tachypnea (respiratory rate 29 breaths per minute). In the absence of apparent local tissue injury, the treating physicians did not attribute the shock state to the snakebite due to the snake species and continued lack of soft tissue injury. They instead pursued an evaluation of alternative diagnoses including vasovagal syncope, arrhythmogenic syncope, cardiac ischemia, hypovolemia, and electrolyte abnormalities while providing supportive care.

One liter of normal saline and atropine 0.5 milligrams (mg) were administered with increase in the heart rate to 68 bpm. However, the patient remained relatively hypotensive (blood pressure ranging from 84–123/54–67 mm Hg) compared to his baseline (blood pressure 138–164/68–74 mm Hg). Electrocardiogram (ECG) showed new submillimeter ST-segment elevations in the anterior precordial leads. Serial troponin T measurements were added to the initial laboratory evaluation and resulted at 0.01 nanograms/milliliter (ng/mL) (reference range: </= 0.10 ng/mL) at both time zero and 12 hours. A consulting cardiologist attributed the dynamic ECG changes to a stress response in the setting of presumed vasovagal syncope. The initial laboratory evaluation collected at the time of patient arrival to the ED was remarkable for a creatine evaluation to 1.4 milligrams per deciliter (mg/dL) (reference range: 0.6 to 1.3 mg/dL) from 1.1 mg/ dL just one month earlier. Platelets were marginally elevated at 523 × (150–450 × 10^9^ per liter [L]). Activated partial thromboplastin time was negligibly low at 25.5 seconds (26.8–37.1 seconds). Prothrombin time was 11.8 seconds (9.5–13.1 seconds), fibrinogen was 334 mg/dL (275 mg/dL), and creatine kinase was 86 units/L (U/L) (30–220 U/L).

The patient received an additional three liters of normal saline resuscitation. He was then observed to have developed ecchymosis, edema, and tenderness nine hours after the time of the initial snakebite. His symptoms were then attributed to envenomation, and crotaline polyvalent immune fab (ovine) antivenom (FabAV) was ordered. Initial control was achieved with six vials. His systemic symptoms and relative hypotension resolved, his blood pressure remained at his baseline afterward, and the leading edge of his tissue edema stopped progressing (Figure). Pain and swelling extended to just below the knee at its worst, and creatinine peaked at 2.0 mg/dL. Five additional two-vial doses of FabAV were administered to maintain control of his local tissue findings. In total, 16 vials of FabAV were administered.

After 51 hours from presentation, he was discharged with vitals that approximated his pre-envenomation baseline, an improving exam, and a downtrending creatinine. On long-term follow-up, his new baseline creatinine was 1.3–1.5 mg/dL consistent with stage three chronic kidney disease.

**CPC-EM Capsule**

What do we already know about this clinical entity?

*Although rare, copperhead envenomation can cause severe systemic toxicity. Envenomation should be promptly treated with antivenom*.

What makes this presentation of disease reportable?

*We present a case of severe systemic envenomation where diagnosis was delayed and the patient developed stage three chronic kidney disease*.

What is the major learning point?

*Copperhead snakebites can cause severe systemic envenomation even in the absence of early local tissue injury*.

How might this improve emergency medicine practice?

*Early treatment requires prompt diagnosis and emergency physicians should be aware that systemic toxicity can precede or occur in the absence of local tissue injury*.

## DISCUSSION

Pit viper snakebites are the most common venomous snakebites in the US.^1^ Copperhead snakes are less likely to cause severe envenomation syndromes than rattlesnakes, and nearly all symptomatic cases eventually develop signs of local tissue injury.^5^–^7^ However, clinicians must still recognize that these trends are not universal, and overt bleeding, systemic toxicity, and even death have been reported with copperhead snakebite.^2^,^8^

The clinical presentation of snakebite is widely variable because venom is composed of a complex mixture of proteins and peptides that exert symptoms across multiple organ systems. The venom effects are often grouped into local tissue injury, hematologic, cardiovascular, neurologic, gastrointestinal, and pulmonary venom toxicity domains.^9^ Severity does not correlate strongly between domains and the presence of venom effects within one domain cannot be used to predict another.^9^ Our patient presented with early systemic symptoms that culminated in hemodynamic instability and syncope despite normal coagulation studies and only isolated fang marks to the affected extremity.

This patient met criteria for antivenom therapy early in his ED visit according to the unified treatment algorithm for management of crotaline snakebite.^10^ He did not receive early antivenom therapy because of a delay in the diagnosis of his envenomation syndrome. After discussion with the physicians providing care to this patient, the absence of erythema, edema, and pain upon his arrival played a role in this delay. However, even in the initial absence of local tissue signs, this patient’s chest tightness, shortness of breath, and abdominal pain should have been recognized as early systemic symptoms.

Snake species biased the treating clinicians who did not recognize that toxicity in one venom effect domain does not predict toxicity in another.^9^ They were falsely reassured that copperhead snakebites rarely cause severe systemic envenomation based on previously published case series where no hypotensive events were documented.^4^,^11^ Some authors have used this observation to recommend against routine use of antivenom in copperhead snakebites.^4^ This approach neglects the rare, severe systemic envenomation syndromes that have been described in the literature, resulting in shock requiring antivenom therapy.^12^

Time from envenomation to treatment greater than six hours increases the likelihood of severe systemic toxicity in other pit viper envenomations.^5^ In copperhead snakebite specifically, later treatment results in longer times to recovery.^13^ Our patient was treated with only fluids and atropine and not FabAV for nine hours after the snakebite. He remained relatively hypotensive until receiving standard-of-care therapy with FabAV, at which point his clinical course began to improve. He was left with stage three chronic kidney disease as a potentially preventable complication.

We hypothesize that this patient’s kidney injury was the result of prolonged hypotension. His fractional excretion of sodium was calculated at the time of his worst documented renal function and found to be 0.1%, making prerenal pathology most likely. Direct and immune-complex nephrotoxicity cannot be entirely excluded. However, he did not have evidence of acute tubular necrosis or microscopic hematuria, and pigment nephropathy seems highly unlikely in the absence of rhabdomyolysis or consumptive coagulopathy.

Well-established cognitive errors led to delays in diagnosis and treatment in this case.^14^, ^15^,^16^ The clinicians became anchored to an incorrect diagnosis through triage cueing and diagnostic momentum.^14^ Their judgment fell victim to representative restraint as this rare case did not comprise all features of severe toxicity across multiple domains.^14^ Prevalence perception played a role as it is uncommon for copperhead snakebites to result in severe systemic toxicity.^15^ These classic cognitive errors resulted in a delayed diagnosis and consequently delayed antivenom treatment. Earlier empiric antivenom was further delayed due to omission bias and the focus on nonmaleficence. 14

The exact etiology of such severe systemic findings in a copperhead envenomation patient, given that most patients have mild or no systemic symptoms, is not clear. The cause may have been specific to this individual snake’s venom, although that degree of within-species venom variation would be extreme. There may have been patient-specific factors that caused his symptoms, such as comorbidities like his chronic hypertension or other undiagnosed cardiovascular disease. There may have also been a patient-specific inflammatory response leading to a cascade of downstream cardiovascular sequelae, which has been demonstrated in other pit viper snakebites.^17^ In all likelihood, the presentation was multifactorial and included several of the possibilities described. The possibility of the patient having a mild envenomation and a concurrent, severe, unrelated cardiovascular event at the same time does not seem plausible. Since his severe symptoms preceded Fab AV administration, we conclude that this was not an adverse reaction to the medication.

## CONCLUSION

Although copperhead snakes are less likely to cause severe envenomation than are rattlesnakes, this case highlights an important example of a severe systemic toxicity with delayed diagnosis and treatment, and a long-term complication. Per existing guidelines, all pit viper snakebites should be observed at a minimum, and antivenom treatment initiated once venom effects progress regardless of venom effect domain.^10^

## Figures and Tables

**Figure:**
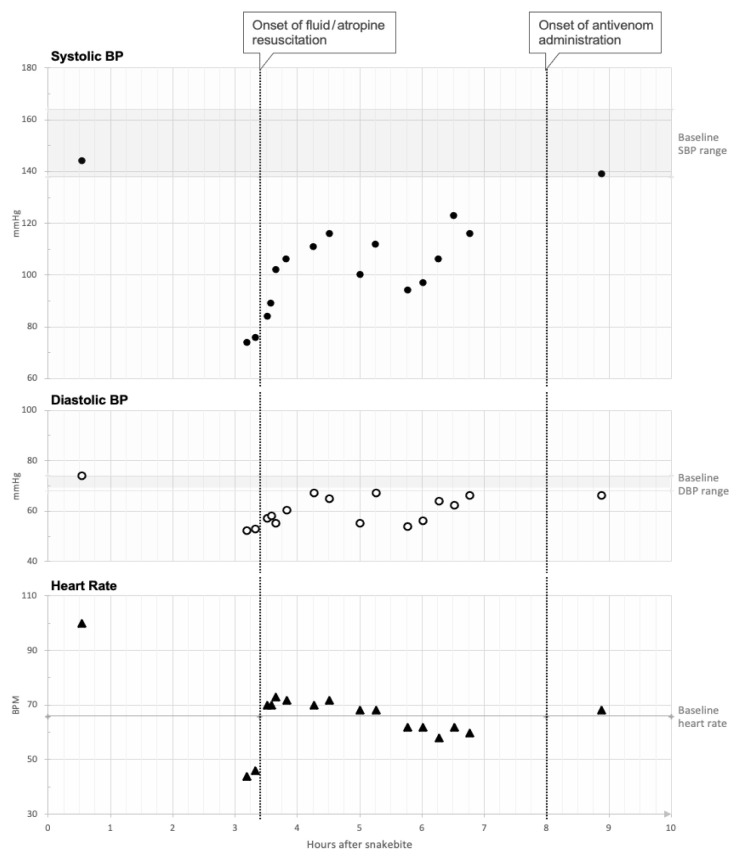
Visual display of the patient’s hemodynamic response to different treatment modalities measured over time. Baseline characteristics (systolic blood pressure, diastolic blood pressure, and heart rate) are depicted with horizontal lines for reference. Onset and type of medical intervention are labeled with vertical, dotted lines. *BP*, blood pressure; *SBP*, systolic blood pressure; *DBP*, diastolic blood pressure; *mmHg*, millimeters of mercury; *BPM*, beats per minute.

**Image:**
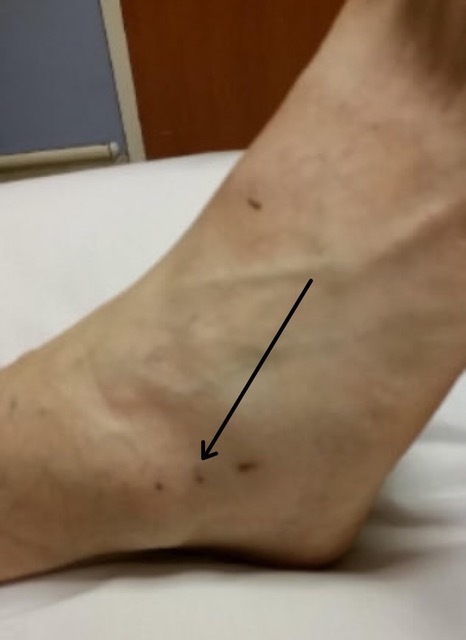
Lateral foot and ankle upon presentation demonstrating puncture wounds (arrow) from a copperhead snakebite without associated local tissue injury.
